# Laparoscopic intervention for late-onset perforating peritonitis due to a ventriculoperitoneal shunt: a case report and literature review

**DOI:** 10.1186/s40792-023-01737-1

**Published:** 2023-09-04

**Authors:** Takeshi Morinaga, Osamu Nakahara, Akira Tsuji, Kunitaka Kuramoto, Masayoshi Iizaka, Shintaro Hayashida, Yuki Ohya, Yasuyuki Hitoshi, Yukihiro Inomata

**Affiliations:** 1 Department of Surgery, Kumamoto Rosai Hospital, 1670 Takehara-Machi, Yatsushiro-City, Kumamoto, 866-8533 Japan; 2 Department of Pediatric Surgery, Kumamoto Rosai Hospital, 1670 Takehara-Machi, Yatsushiro-City, Kumamoto, 866-8533 Japan; 3 Department of Neurosurgery, Kumamoto Rosai Hospital, 1670 Takehara-Machi, Yatsushiro-City, Kumamoto, 866-8533 Japan

**Keywords:** Ventriculoperitoneal shunt, Late-onset generalized peritonitis, Perforation of small intestine, Laparoscopic surgery

## Abstract

**Background:**

Ventriculoperitoneal (VP) shunt placement is commonly performed to treat hydrocephalus and complications are not uncommon. We report here a case of generalized peritonitis caused by migration of the abdominal end of a VP shunt catheter into the bowel after multiple VP shunt revisions over 30 years. Laparoscopic surgery was successfully performed for the peritonitis and the VP shunt system subsequently reconstructed without complications.

**Case presentation:**

The patient was a 49-year-old woman who had a VP shunt placed for obstructive hydrocephalus at the age of 13 years. The shunt system required seven revisions because of various malfunctions, including two occasions where a nonfunctioning shunt catheter was left inside the abdomen for safety reasons. Approximately 1 year after the seventh revision, she developed abdominal pain and fever. Abdominal computed tomography suggested that the shunt catheter had migrated into the small intestine and caused an intra-abdominal abscess. We performed emergency exploratory laparoscopy, which revealed perforation of the small intestine by the tip of a nonfunctioning shunt catheter. A growing abscess was found around the perforated intestinal wall, causing bacterial ascites. After the functioning shunt catheter was pulled out from the abdomen, the nonfunctioning catheter that had perforated the intestinal wall was removed. The functioning shunt catheter was then connected to the cerebrospinal fluid drainage system to manage her severe hydrocephalus. Finally, the contaminated abdominal cavity was copiously irrigated with saline solution and a peritoneal drain placed. Twenty-five days later, she underwent another VP shunt surgery in which a VP shunt catheter was placed. She was discharged 45 days after the surgery for peritonitis without complications.

**Conclusion:**

In cases of peritonitis with a history of VP shunt placement, perforation by a VP shunt catheter is possible, though rare. A delay in treatment could lead to a potentially fatal complication, such as septic shock. Laparoscopic surgery enabled a faster, more hygienic, and minimally invasive operation for managing this rare but serious complication of VP shunt placement.

## Background

Ventriculoperitoneal (VP) shunting is widely used to treat hydrocephalus, but is associated with various complications, including intra-ventricular complications such as infections, catheter bending or obstruction, intestinal obstruction, and injury to intra-abdominal organs [1–4]. Gastrointestinal perforation is relatively rare, with a reported incidence of 1.0% [5]. In addition, most cases of gastrointestinal tract perforation have been reported in children, with much shorter intervals between surgery and symptom onset than in adult cases.

We report here a case in which a peritoneal shunt catheter migrated into the small intestine leading to generalized peritonitis 30 years after the initial VP shunt surgery. Laparoscopic surgery was successful, and the patient recovered without complications.

## Case presentation

The patient was a 49-year-old woman. At 13 years of age, she developed acute obstructive hydrocephalus and underwent VP shunt surgery. She subsequently underwent seven shunt revisions due to various shunt complications. On two occasions, the peritoneal portion of the catheter was left in the abdomen for safety reasons. Most recently, a shunt catheter was placed into the left abdominal cavity via the left temporal and thoracic wall when she was 48 years old.

**Fig. 1:**
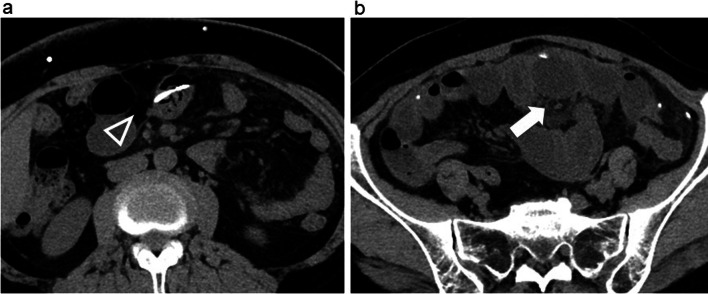
**a** CT scan showing that the tip of the right shunt catheter had entered the lumen of the small intestine (white arrowhead). **b** CT scan showing abscess formation on the caudal side of the perforating catheter in the small intestine (white arrow)

The following year, she visited our hospital for a routine exam. Physical examination and blood test findings were normal. However, she returned just 3 days later with abdominal pain and fever. Physical examination revealed peritoneal irritation. Computed tomography (CT) suggested migration of a shunt catheter into the lumen of the small intestine in the right side of the abdomen with an enlarging abscess cavity (Fig. 1a, b).

**Fig. 2:**
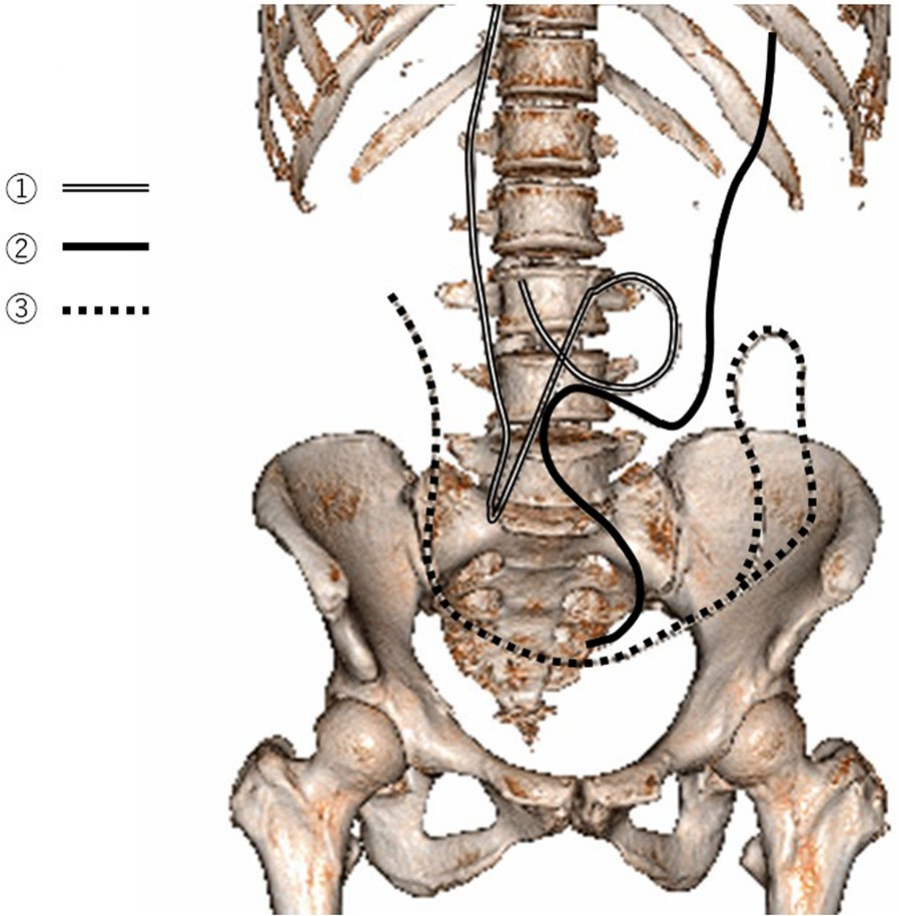
3D reconstruction of CT scans of the VP shunt catheters. ① Nonfunctioning shunt catheter (double solid black line) in right side of the abdominal cavity: responsible for intestinal penetration. ② Functioning shunt catheter (solid black line) in the left side of the abdominal cavity: the tip is in the pelvic cavity. ③ Shunt catheter disconnected at both ends (black dotted line) abandoned in the abdominal cavity during a previous reconstruction: both ends were floating in the peritoneal cavity

We performed an emergency exploratory laparoscopy suspecting acute peritonitis associated with intestinal perforation caused by one of the shunt catheters. CT results and detailed records from her multiple surgeries indicated that there were three separate shunt catheters in her abdomen at the time of surgery: a functioning shunt catheter on the left side, a nonfunctioning catheter in the right abdominal cavity left during a previous reconstruction 6 years earlier, and another nonfunctioning catheter in the pelvic cavity from the third surgery (Fig. 2). One of the nonfunctioning catheters was responsible for the intestinal perforation.

Prior to laparoscopic intraperitoneal observation, we pulled the functioning catheter out from the abdominal cavity to place an external drain for the cerebrospinal fluid (CSF). We then inserted a laparoscope through the umbilicus and observed the abdominal cavity. Next, we removed the free migrated shunt catheter found in the pelvic cavity. Finally, we removed the catheter that had perforated the intestine, and the perforation was sutured and closed with 3-0 Vicryl. The peritoneal cavity was irrigated with 10 L of warm saline, and a 19-Fr Blake drain was placed.

Postoperatively, *Enterobacter cloacae* was detected in both ascites and CSF culture, and meropenem plus vancomycin was administered to control the infection. A liquid diet was started on postoperative day 5. The CSF gradually became clear, but bacterial cultures remained positive even 2 weeks after the operation. After confirming that the CSF became aseptic, we constructed a new VP shunt on postoperative day 25. First, the neurosurgery team removed the functioning VP shunt system and placed the new one in the ventricular cavity. The gastrointestinal surgery team then dissected severe adhesions of the abdominal wall laparoscopically and secured a space for a shunt catheter in the pouch of Douglas to complete the reconstruction.

Postoperatively, antibiotic treatment was continued as prophylaxis against meningitis. Fortunately, there were no signs of infection, and she was discharged home 45 days after the surgery for peritonitis.

## Discussion

VP shunt placement is the preferred procedure for treating hydrocephalus. Various complications of VP shunting have been reported to occur in 24–47% of cases [1, 6], including shunt catheter migration to various parts of the body such as the mouth, internal jugular vein, heart, lung, pulmonary artery, and thoracic cavity [2–4, 7–20]. In some cases, the shunt catheter has even been reported to perforate the gastrointestinal tract [21]

If shunt malfunction requires the shunt system to be removed, the initial step must be to examine for signs of bacterial infection. If there is no accompanying peritonitis or abdominal abscess, the abdominal catheter can simply be pulled out percutaneously without inspecting inside the abdomen. However, if there are any signs of bacterial infection inside the abdomen, perforation of the gastrointestinal wall by the shunt catheter should be suspected.

In most previous cases, the site of gastrointestinal wall perforation usually has a thick fibrous membrane blocking the leakage of bowel content [22]. As a result, the patient might remain asymptomatic even after perforation has occurred. If the perforation causes a local abscess or generalize peritonitis, the leakage site might not heal spontaneously, and surgical intervention is the only option for successful treatment.

Laparotomy is generally the preferred method in cases where the exact location of the gastrointestinal wall perforation is difficult to determine on imaging. Alternatively, if a preceding examination can predict the possible location of the perforation, then laparoscopy is a better method as it is minimally invasive and allows the site of interest to be reached more quickly. We chose exploratory laparoscopy in this case since preoperative CT identified the possible perforation in the small intestine. Intra-abdominal irrigation is essential to eliminate bacterial contamination, especially in cases of severe peritonitis caused by the gastrointestinal perforation. Laparoscopic surgery is suitable for this procedure because the system is already equipped with an efficient irrigation apparatus that can easily wash out contaminants with a high flow of the sterile solution not only locally but throughout the whole abdominal cavity [23]. Another benefit of laparoscopy is the minimal insult to the peritoneum, which can help prevent adhesive peritonitis that impedes absorption of water from the shunt catheter. Even in cases without infection, laparoscopy has become a popular alternative for accessing the peritoneal cavity because it is less time consuming, more hygienic, and minimally invasive [24, 25].

**Fig. 3:**
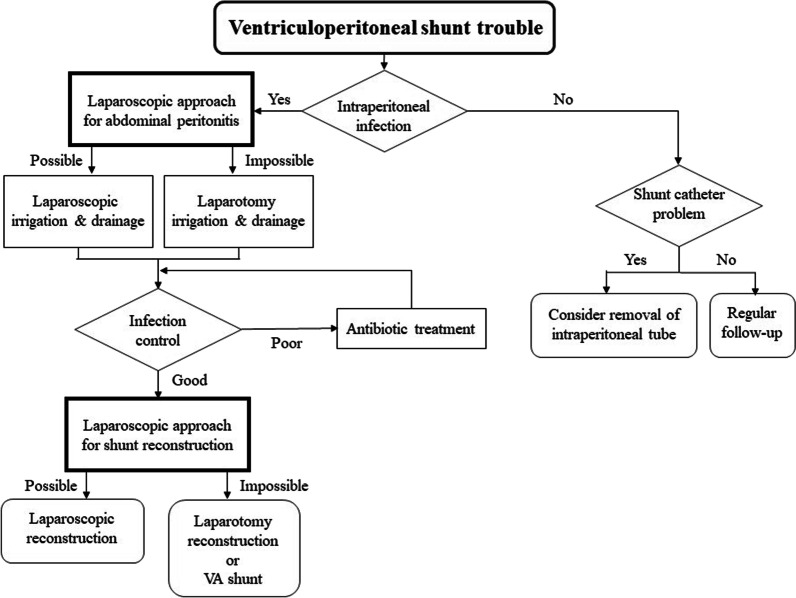
Algorithm for handling ventriculoperitoneal shunt trouble at our institution

**Fig. 4:**
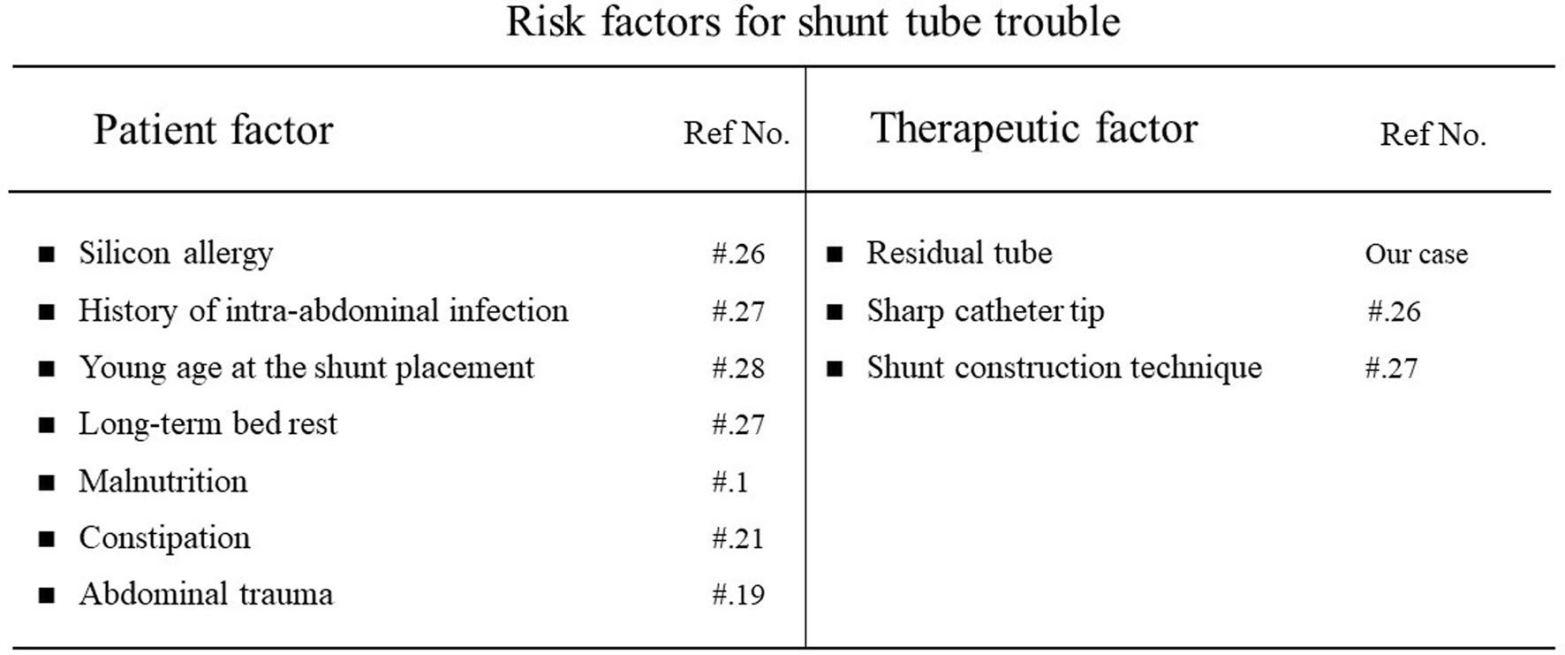
Risk factors for shunt catheter problems

We tried to create an algorithm for handling ventriculoperitoneal shunt trouble at our institution (Fig. 3). Although accidental perforation can occur, the shunt catheter itself is made of a silicone-based material with a special coating, which was originally designed not to injure any structures within the abdomen. Although the precise cause of perforation is not reported in most cases, one case study suggests that the following factors might contribute to gastrointestinal perforation: trocar use for catheter insertion, a sharp tip of the shunt catheter used, and the duration that the catheter is indwelling [26]. Other reports suggest silicone allergy, history of intra-abdominal infection, malnutrition, and long-term bed rest as risk factors for gastrointestinal perforation [27, 28] (Fig. 4). In our case, the exact cause of gastrointestinal perforation is not known. The history leading up to the present incident was as follows. The shunt catheter responsible for the perforation was placed in the sixth VP shunt reconstruction performed 6 years earlier. During the surgery for the seventh VP shunt reconstruction 1 year before the present incident, slight resistance was felt when attempting to remove the ventricular catheter from the sixth VP shunt system, and the removal of that shunt system was stopped to avoid catastrophic complications such as intra-ventricular hemorrhage. Considering the severe nature of the patient’s obstructive hydrocephalus, and the fact that the sixth shunt system was still partly functioning, the entire old sixth shunt system was kept in place. Ultimately, the seventh shunt system was constructed safely on the contralateral side, thus creating double shunting.

There are alternative ways to place the end of the shunt catheter, with a ventriculoarterial shunt (VA) as one option. Unfortunately, the neurosurgeon working at that time had no experience with VA shunt placement. In addition, considering the risk of contamination and thrombosis, we preferred continuing to use the abdomen for CSF absorption.

Shiro et al. have presented a wide variety of possible mechanisms causing gastrointestinal perforation, from simple physical pressure of the tip of the catheter to the unintuitive idea of CSF droplets causing a water-hammer effect [20]. However, in our case, the symptoms appeared after obstruction of the CSF flow had been detected, ruling out the water-hammer effect. Another plausible explanation is gradual erosion of the wall structure by chronic inflammation due to some kind of immune response to the silicone material, assuming the tip of the shunt catheter is permanently anchored to soft tissue adjacent to the gastrointestinal wall. Regardless of the pathology of gastrointestinal perforation, long-term indwelling of the catheter inside the abdominal cavity is an inherent risk factor for a wide range of problems. To avoid unnecessary risk of infectious complications, if the CSF shunt system malfunctions, removal of at least the abdominal catheter should be attempted if the safety of the procedure can be ensured. However, there are some cases of shunt malfunction where the catheter cannot be safely removed. In such cases, if there are any signs or symptoms suggesting bacterial peritonitis, injury to the gastrointestinal wall causing leakage of bowel contents should be suspected.

## Conclusions

Gastrointestinal perforation by an abdominal shunt catheter is a rare complication that can occur after VP shunt surgery, even when the catheter is no longer functioning. In our case, this complication could be treated safely by laparoscopic surgery, which involved removal of the perforating catheter, closure of the gastrointestinal perforation, irrigation of the peritoneal cavity, and placement of another VP shunt catheter. Laparoscopy minimized injury to the peritoneum, preserving the capacity to absorb CSF.

## Data Availability

Not applicable.

## Abbreviations

VP shunt: Ventriculoperitoneal shunt
CT: Computed tomography
CSF: Cerebrospinal fluid
